# Draft Genome Sequence of the Plasmid-Free *Lactococcus lactis* subsp. *lactis* Strain LMG 19460

**DOI:** 10.1128/genomeA.00210-17

**Published:** 2017-04-20

**Authors:** Inês N. Silva, Sofia Duarte, Leonilde M. Moreira, Gabriel A. Monteiro

**Affiliations:** a Institute for Bioengineering and Biosciences, Instituto Superior Técnico, Universidade de Lisboa, Lisboa, Portugal; b Department of Bioengineering, Instituto Superior Técnico, Universidade de Lisboa, Lisboa, Portugal

## Abstract

We report here the draft genome sequence of the plasmid-free *Lactococcus lactis* subsp. *lactis* strain LMG 19460. This strain has potential application for a cost-effective production of food-grade plasmid DNA to use in DNA vaccines, produce recombinant proteins, and be used as a mucosal delivery vehicle of therapeutic molecules.

## GENOME ANNOUNCEMENT

Lactic acid bacteria (LAB) have a huge relevance in health, food, and industrial applications, being used traditionally as fermentation starters and recently as probiotics (1), mucosal delivery vehicles of therapeutic molecules (2), and recombinant protein producers (1).

*Lactococcus lactis* is the model LAB, being broadly used in the dairy industry (3). The availability of several molecular biology tools to manipulate this bacterium, together with its food-grade and generally recognized as safe (GRAS) status, raises even more the industrial value of this species (2).

We report here the genome sequence of *L. lactis* subsp. *lactis* LMG 19460, which is a plasmid-free strain, formerly known as *Streptococcus lactis* subsp. *diacetylactis* Bu2-60. This strain is derived from *S. lactis* subsp. *diacetylactis* Bu2 that was exposed to high sublethal temperatures to cure its six plasmids (4–6). The parental strain was isolated from starter cultures of German cheese factories between 1971 and 1979 (7).

Although the LMG 19460 strain lacks the plasmid-coded metabolic functions such as carbohydrate and citrate metabolism, which make it infeasible to be used as a starter culture in dairy fermentation, it has several advantages. This strain is ideal for DNA transfer and gene cloning studies, since issues due to the presence of other plasmids of the same compatibility group are avoided, as well as heterologous DNA degradation problems derived from the presence of two plasmid-encoded restriction-modification systems (8). Studies have been performed using this strain as a recipient for conjugation (4, 9, 10) and for plasmid transfer via transduction (11).

A less explored application of this strain is its potential as a host for the production of food- and pharmaceutical-grade plasmids for use in DNA vaccines (12, 13). Together with being GRAS, the plasmid-free status of this strain allows a cost-effective purification process, due to the absence of copurifying endogenous plasmids and/or pathogenic contaminants (12). Also, the decrease in the metabolic burden could lead to an increase in the plasmid yields and also in the production of pharmaceutical-grade recombinant proteins.

Whole-genome sequencing of *L. lactis* subsp. *lactis* LMG 19460 was performed using the Illumina MiSeq platform (Instituto Gulbenkian de Ciência, Portugal), which yielded a total of 753,312 paired-end reads (totaling ~140× coverage). Paired-end reads were analyzed for Phred quality, trimmed and filtered using Fastq-Mcf version 1.04.676 (14), and assembled using SPAdes version 3.8.0 (15) and HGA version 1.0 (16). Generated contigs were scaffolded using SSPACE version 3.0 (17) followed by automated improvement using iCORN (18). The *L. lactis* subsp. *lactis* LMG 19460 genome was assembled in 41 contigs accounting for 2,260,841 bp and an estimated G+C content of 35.1%. The draft genome was annotated using the NCBI Prokaryotic Genome Annotation Pipeline, which predicted a total of 2,164 protein-coding sequences, 84 pseudogenes, six rRNAs, and 51 tRNAs.

Further experimental and comparative genomic analyses will provide new insights into the use of this strain to produce recombinant proteins and as a delivery vehicle for therapeutic molecules.

### Accession number(s).

This whole-genome shotgun sequence project has been deposited at DDBJ/ENA/GenBank under the accession number MUBH00000000. The version described in this paper is the first version, MUBH01000000.
